# Accelerating HPV vaccination in Africa for health equity

**DOI:** 10.1186/s41256-024-00380-z

**Published:** 2024-09-19

**Authors:** Eric Asempah, Ene Ikpebe

**Affiliations:** 1 Dahdaleh Institute for Global Health Research, York University, 4700 Keele Street, Toronto, Canada; 2 Askew School of Administration and Policy, South Florida University, 113 Collegiate Loop, Tallahassee, FL 32306 USA

**Keywords:** Regionalization, Africa, HPV, Vaccination, Cervical cancer, Health equity

## Abstract

Cervical cancer is a preventable disease that continues to burden socioeconomically underserved regions, especially in Africa. Vaccination of adolescents who have never had sex with prophylactic human papillomavirus (HPV) vaccines proves effective in preventing the disease. However, vaccine accessibility and availability are two persistent challenges in low-resource settings. For this commentary, a trend analysis is conducted for national HPV vaccination and coverage rates in Africa, a region with high burden of the disease. This is in consideration of the World Health Organization (WHO) strategy to vaccinate 90% of adolescent girls by the age of 15, as part of strategy to eliminate cervical cancer by 2030. The analysis estimated that the rate of incorporating HPV vaccination in national immunization programs in Africa occurs slowly, at a mean wait time of 12 years with estimated coverage rate of 52%. A policy change that harnesses strategic approaches, such as a regionalized vaccination program, is recommended to hasten HPV vaccination for the rest of African countries without a national program.

## Background

About 604,000 new cases and 342,000 deaths due to cervical cancer occur globally per annum. Of this, it is estimated that 117,316 women in Africa are diagnosed, of which 76,745 die from the disease every year [1]. In response to this public health issue, the World Health Organization (WHO) made a firm commitment in 2018 to eradicate cervical cancer by 2030, ensuring that 90% of girls are vaccinated against HPV before the age of 15, by screening 70% of women aged 35–45 for cervical cancer, and by treating 90% of affected women globally [2]. The WHO strategy is particularly relevant because while over 200 different strains of HPV exist, more than a dozen strains have been identified as high-risk (hrHPV) which are linked to 90% of all cervical cancer as well as various types of other cancers (e.g., vulva, vagina, anus, penis, and oropharynx). Also, of these, strains 16 and 18 have been identified as an etiological cause of cervical cancer. Prophylactic HPV vaccines exist for the prevention of HPV-related cervical cancer when administered prior to exposure to the cancer-causing hrHPV. However, access to these preventative vaccines is either non-existent or challenging in some low- and middle-income countries (LMICs), a situation not found in high-income countries (HICs) where proactive public policy and responsive governmental priority settings ensure that HPV vaccines are readily available and accessible. In a comprehensive review by Dutta, Meyerson, and Agley, it revealed that only 12 out of the 54 African countries (22.2%) have established plans to tackle cervical cancer. Alarmingly, the plans from 8 of these 12 countries (69.2%) were ineffective due to program expiration [3]. We noted that in many cases, these countries had integrated their cervical cancer prevention plans into broader disease-focused strategies, potentially limiting the impact of dedicated interventions. Evidence shows that places with low Human Development Index (HDI) have disproportionately higher levels of cervical cancer incidence and mortality [4]. This relationship between HDI and cervical cancer cases raises concern for Africa, where HDI has been historically low with high Human Poverty Indices (HPI), and only 1–2% of women between the age of 10–20 having received HPV vaccination [5]. The International Papillomavirus Society (IPVS) has declared support for prioritizing WHO’s 90% vaccination for “sexually naïve girls before age 17 by 2030,” which must revolve around equitable access to the vaccine [6].

Three vaccines that have dominated the market, Gardasil^®^ and Gardasil^®^9 by Merck, and Cervarix^®^ by GSK, required multiple doses, leading to significant cost implications to governments especially in LMICs. However, recently approved single-dose vaccine, Cecolin^®^ by Xiamen, provides equivalent efficacy to the forerunner HPV vaccines at reduced cost. A cost-effectiveness study comparing the three vaccines demonstrated that Cecolin^®^ provides “lowest net cost and the most attractive cost-effectiveness” [7]. New vaccine entrants such as Cecolin^®^ can revolutionize HPV vaccination in LMICs and represents a significant opportunity for African leaders to proactively engage with manufacturers and key stakeholders from the outset.

Accelerating access to HPV vaccines in Africa must be a priority for policymakers to lessen the burden and mortality rate of cervical cancer in the region. This will require policy changes and proactive updates to existing strategies for intervention. The first crucial step is to analyze the current national vaccination efforts in the region, as this can provide valuable insights into the need for these changes. To do this, a list of countries in Africa with a national HPV vaccination program is compiled and assessed to establish a baseline, using information from https://hpvcentre.net/datastatistics.php. The list is crosschecked using Google search for HPV vaccination in each country in the region, and where available online, the country health report is searched for corroboration.

## HPV vaccination trend and coverage rate

As of 2023, 29 of the 54 African countries include HPV vaccination in their national immunization program. Even though the first HPV vaccine Gardasil^®^ (manufactured by Merck) became publicly available in 2006, it took five years for the first African country, Rwanda, to incorporate the vaccine in its national immunization program in 2011.

Comparing the timelines of the 29 African countries with a national HPV vaccination program (Table 1), it takes on average 12 years for an African country to introduce the vaccine in national immunization program. With a coefficient of variation of 33.3%, moderate variability in the wait time for the vaccine implementation exist. Given the current trend, adoption of national HPV vaccination program covering the entire region is projected to be 2039. The WHO’s elimination target of 2030 is estimated to be 9 years short of the current trend of the vaccine implementation in Africa. New or enhanced strategies are needed to hasten vaccination and catalyse the slow implementation trend.

**Table 1:** Descriptive statistics of African countries with some form of national HPV vaccination program

African country	Cohort	Year of HPV vaccine 1st introduction	Wait time for HPV vaccination implementation (years)^a^	Standard deviation	Variance	National Vaccination coverage rate^b^			
						First dose (%)	Last dose (%)	Average dose coverage	Year of estimation
HPV Vaccine Availability	For Males and Females	2006	0	0	0	N/A	N/A	N/A	N/A
Rwanda	Females	2011	5	2.5	6.25	78	73	76	2021
Lesotho	Females	2012 (reintroduced in 2022)	6	2.6	6.88	68^c^^,d^	60^c^^,d^	64	2022
Libya	Females	2013	7	2.7	7.25	96^d^	N/Av	96	2021
South Africa	Females	2014	8	2.8	7.76	37	34	36	2021
Seychelles	Females	2014	8	2.8	7.56	84	39	62	2021
Uganda	Females	2015	9	2.8	7.84	75	44	60	2021
Botswana	Females	2015	9	2.8	7.75	N/Av	22	22	2021
Mauritius	Females	2016	10	2.8	8.01	78	55	67	2021
Ethiopia	Females	2018	12	3.1	9.64	86	75	81	2021
Senegal	Females	2018	12	3.2	10.51	39	21	30	2021
Tanzania	Females	2018	12	3.3	10.97	73	57	65	2021
Zimbabwe	Females	2018	12	3.3	11.17	67	40	54	2021
Côte d’Ivoire	Females	2019	13	3.4	11.74	34	41	38	2021
Gambia	Females	2019	13	3.5	12.06	34	30	32	2021
Kenya	Females	2019	13	3.5	12.21	29	44	37	2021
Liberia	Females	2019	13	3.5	12.25	43	30	37	2021
Malawi	Females	2019	13	3.5	12.20	14	12	13	2021
Zambia	Females	2019	13	3.5	12.09	45	33	39	2021
Cameron	Males and Females	2020	14	3.5	12.29	5	20	13	2021
Cabo Verde	Females	2021	15	3.6	12.79	90	N/Av	90	2021
Mauritania	Males and Females	2021	15	3.6	13.16	39	N/Av	39	2021
Mozambique	Females	2021	15	3.7	13.41	57	N/av	57	2021
Eritrea	Females	2022	16	3.7	13.95	99^d^	99^c d^	99	2023
Sierra Leone	Females	2022	16	3.8	14.37	N/Av	N/Av	-	-
Morocco	Females	2022	16	3.8	14.69	N/Av	N/Av	-	-
Burkina Faso	Males and Females	2022	16	3.9	14.92	N/Av	N/Av	-	-
Eswatini	Females	2023	17	3.9	15.42	N/Av	N/Av	-	-
Nigeria	Females	2023	17	4.0	15.82	N/Av	N/Av	-	-
Togo	Females	2023	17	4.0	16.13	N/Av	N/Av	-	-
			Mean = 12	SD = 4.0	Coefficient of Variation (4.0/12) × 100 = 33.3%	Mean = 57.72	Mean = 43.63	Mean = 52.47	

^a^The wait time for HPV implementation is how long a country takes to implement a national vaccination program from when the vaccine became commercially available in 2006

^b^Source (unless otherwise stated) https://hpvcentre.net/statistics/reports/ZMB_FS.pdf?t=1722621948434

^c^Data obtained for program reintroduction in 2022

^d^source https://immunizationdata.who.int/global/wiise-detail-page/human-papillomavirus-(hpv)-vaccination-coverage?CODE=LSO&ANTIGEN=HPV_FEM&YEAR =

*N/Av* Not available

The data (Table 1) also show that HPV vaccination coverage in Africa is low, with only about 52% coverage for the first and last dose, based on available data. This indicates significant challenges to vaccine coverage and uptake, stemming from various factors, including last-mile delivery challenges due to poor road infrastructure-especially in rural areas, inadequate information about the vaccine’s efficacy and safety, and cultural sensitivities to vaccination-leading to hesitancy. However, it is important to note that some African countries, such as Rwanda, Ethiopia, and Mauritius, have achieved exceptionally high vaccination coverage rates of over 70%, in alignment with the WHO’s strategy to eliminate cervical cancer. Increasing public education campaigns on vaccine safety and engaging local community and opinion leaders to participate actively is expected to drive uptake and coverage rates.

## Priority setting for action

The initially high cost of the HPV vaccines from its onset gave many LMICs a reason to shift away from engaging in the rush for the vaccine in the early stages of the vaccine’s commercialization. In 2011, the price of HPV vaccine dropped to a record low for developing countries ($ 4.50 USD per dose) when it was over $100 USD in HICs [8]. This made it possible for some African countries to reconsider their cervical cancer prevention program and policy. Around this period, Rwanda took advantage of the market situation and through negotiation with Merck became the first country in Africa to roll out a nationwide HPV vaccination program for girls. Rwanda received 2 million doses of Gardasil^®^ vaccines donated from Merck for a period of three years, as a starter-pack for its program [9]. After the three-year arrangement with Merck was about to end, Gavi intervened to continue the arrangement with Merck to keep the supply of HPV vaccine to Rwanda until 2017.

In many African countries, priority setting and resource allocation are challenged by competing governmental interests, wherein diseases causing higher mortality often are given preference. This situation is further challenged by fragmented policy actors, driving low political will, and weak health systems contributing to policy inertia in the region. The reflex implication is a continued delay in national HPV vaccination for adolescents due to low prioritization of cervical cancer and resource allocation, if any at all. Achieving low cervical cancer incidence and mortality would demand governmental priority setting and strategic plans for active interplay of internal and external stakeholder engagement. Rwanda shows how governments in Africa with minimal resources and significant competing health needs can prioritize and mobilize internal and external support for a national vaccination program [10, 11].

While Gavi’s support is crucial for African nations that qualify for vaccine purchase assistance, it is important to note that countries that do not qualify may face donor/support inequity. This is particularly concerning for diseases with low priority and resource allocation, where policy entrepreneurs are less visible and the required political will for policy change is lacking. In such cases, policymakers may be inclined to maintain the status quo, which could significantly delay the process of disease elimination by 2030. Merck’s Gardasil Access Program (GAP) previously helped LMICs obtain the vaccines. However, Merck has now ended the program, limiting access to the vaccine for LMICs that do not qualify for Gavi support and cannot implement a national vaccination program. It is estimated that Merck could reduce the cost of the vaccine from $4.50 per dose (the cost to Gavi) to about $1 or even lower [12]. Lowering the cost could make the vaccine more accessible and encourage governments to collaborate with donors or self-finance vaccine procurement for their national vaccination programs, creating shared social benefits.

Vaccine manufacturers have in the past few years faced production challenges, which makes it difficult to meet the market demand. As a result, in 2019, the WHO Strategic Advisory Group of Experts on Immunization recommended that all countries temporarily suspend vaccination plans targeting boys, older age groups (> 15 years), and multi-age cohorts (MAC) [13]. This recommendation aimed at ensuring equitable access, particularly for countries most in need of the vaccine. Even though commercialization of Cecolin^®^ promises to close the supply gap, the heightened demand for the vaccine and competing manufacturing demands, makes it increasingly unlikely that the WHO’s elimination target can be achieved. New HPV vaccine candidates are in clinical phases, however,  and are likely to take years to be licensed for commercial production.

Moving forward, supplier channels should effectively be expanded to close any raw material shortage gaps, expedite production using third-party contract manufacturing organizations (CMOs) to fill the vaccine scarcity gap, and ensure equitable access to the vaccine through proactive programs and policies. Governments in low-resource settings such as Africa that are unable to access external support for a national vaccination program need to be innovative with minimal resources and leverage efficient solutions to public health problems. For example, regionalization of vaccination programs is one approach to hasten HPV vaccination. COVID-19 has taught us few lessons, especially with the COVID-19 Vaccine Global Access (COVAX) facility designed to assure global equitable access to the COVID-19 vaccines. Whereas COVAX’s strategy to reach underserved regions with COVID-19 was challenged and constraint with ethical, management, financing, and supply issues, the principles undergirding the framework remain relevant and can benefit vaccine equity [14]. In operationalizing a regionalized strategy, the African Union Center for Disease Control (CDC) must take a frontline role and ensure logistical systems and frameworks are in place to support stakeholder engagement, prudent purchase, and equitable distribution of vaccines to countries in the region. This should encompass a comprehensive plan that outlines specific steps and measures aimed at achieving vaccination coverage rate that exceeds 70%. While external donor support has been instrumental in making vaccines accessible and available in most LMICs, domestic sources of funding and mobilization of resources must be explored and harnessed, inter alia. These strategies are pivotal approaches to increase access to the vaccines, thereby reducing disparities in health outcomes.

## Conclusions

Considering the current HPV vaccination trend and coverage rate in Africa, it is evident that WHO’s cervical cancer elimination strategy by 2030 is far from being achieved unless drastic policy changes in the region occur. Six suggestions are recommended to: (1) accelerate the current vaccination trend in Africa and other underserved regions where challenges exist in access to HPV vaccines, (2) incentivize vaccine manufacturers to increase production and distribution of the vaccine and make it available, affordable, and accessible to low resourced regions, (3) incorporate a regionalized strategy of vaccine purchasing system and distribution wherein low-resource countries can benefit without overburdening an already overstretched and in most cases, low health expenditure budget, (4) design culturally tailored community-based interventions to reach marginalized and vulnerable communities coupled with monitoring and evaluation to identify gaps and inequalities in vaccination coverage, (5) leverage the influence of men by involving them in immunization as part of a comprehensive and equity focused strategy to combat HPV and cervical cancer, and (6) develop sustainable financing of vaccination through incremental shift to domestic and continental funding sources. In conclusion, the existing inequity expressed in the slow implementation of national HPV vaccination program and relatively low coverage rate in the region highlights a crucial challenge to health equity. It is imperative to engage all stakeholders to reform the current approach and reduce HPV and related diseases, especially, cervical cancer, across the region.

## Data Availability

All data generated or analysed during this study are included in this published article.
